# Botulism in Bihor County during 2002-2014

**DOI:** 10.1186/1471-2334-14-S7-P68

**Published:** 2014-10-15

**Authors:** Balog Eniko

**Affiliations:** 1 Municipal Clinical Hospital "Dr. Gabriel Curteanu" Oradea, Romania

## Background

Botulism is a food-borne disease caused by botulinum toxin produced by *Clostridium botulinum*, under anaerobic conditions. Toxin type B is responsible for producing the disease more often.

## Methods

We conducted a retrospective study of cases of botulism admitted to the Infectious Diseases Department I and II in Oradea during 2001-2014, July. All patients in the study were diagnosed with food poisoning by *Clostridium botulinum*, the food most commonly involved being smoked pork bacon, homemade and untreated by heat. Diagnosis was based on case history data, clinical and toxin identification (often late).

## Results

During 2001-2014 (July) in Bihor County were confirmed 61 cases of botulism food, aged between 5 and 69 years, 57.37% were from rural areas. Homemade pork preparation was most often responsible for transmitting the disease, including the last three years. The highest incidence was in 2004, 2007 and 2012 (1.9 per 10,000 population). Generally sporadic cases were recorded and family outbreaks. In the last three years, there have been 1/3 of the total confirmed cases (20 cases) of botulism, one third of which were severe, but no deaths. Incubation was between 24 hours and 5 days, but some patients arrived at the hospital even after 7 or 14 days after the first symptoms, only 18 patients (35.29%) being hospitalized in the first week of illness. Admission to our department was in most cases by reference from other specialties - neurology and ophthalmology, but also neurosurgery, otolaryngology (2 cases), surgery (2 cases) and psychiatry (1 case). Specific therapy represented by serum specific antibotulinic (botulinum antitoxin) was administrated in 57 cases (93.44%), initially serum polyvalent A, B, E, and in recent years the serum antibotulinic - type F 10,000 IU/f, B 5000 IU/f and type E 10,000 IU/f. Routinely we administered 1 or 2 ampoules/im or iv after preliminary testing and desensitization. In all cases we administered iv ampicillin and metronidazole orally.

## Conclusion

Specific treatment contributed to the favorable development, but specific serum unavailability this year, may cause development of other cases.

